# Circumferential Damage Monitoring of Steel Pipe Using a Radar Map Based on Torsional Guided Waves

**DOI:** 10.3390/s23218734

**Published:** 2023-10-26

**Authors:** Zhupeng Zheng, Zihao Zhang

**Affiliations:** 1 Department of Civil Engineering, Xiamen University, Xiamen 361005, China; shallowsing95@gmail.com; 2 Shenzhen Research Institute of Xiamen University, Shenzhen 518087, China

**Keywords:** torsional guided wave, damage monitoring, circumferential positioning, radar map, sensitivity analysis

## Abstract

Ultrasonic guided wave technology has been successfully applied to detect multiple types of defects in pipes. However, the circumferential location and coverage of a defect are less studied because it is difficult to determine. In this study, the fundamental torsional mode T (0, 1) is selected to conduct monitoring of the circumferential defect in pipelines because of its almost non-dispersive property. A radar map of the peak wave signals at 30 circumferential positions is proposed to detect the damage. The circumferential defect of a steel pipe is thoroughly investigated using numerical simulation. First, the circumferential positioning of defects in various areas of the pipe is studied. Second, the results are compared to those based on longitudinal guide waves. Finally, the circumferential coverage of a defect in the pipeline is determined. The waves are excited and received using the pitch–catch approach, and the collected monitoring signals are processed using the Hilbert transformation. According to the findings, the circumferential defect in the pipe can be effectively identified from a ‘T’ shape in the radar image, and the monitoring method by the torsional guided wave is superior to the longitudinal wave method. The results clearly demonstrate the advantages of torsional guided waves in defect monitoring. The proposed method is expected to provide a promising solution to circumferential damage identification in pipelines.

## 1. Introduction

With the extension of service life in oil and gas pipelines, various defects or damages occur gradually. Ultrasonic guided wave detection technology has the advantages of long propagation distance, low attenuation, and large detection range compared to traditional methods, such as magnetic flux leakage, eddy current, and X-ray [1,2,3]. Therefore, it has been widely used in structural health monitoring (SHM) and non-destructive testing (NDT) of pipeline structures [4,5,6,7,8].

The longitudinal guided wave mode of L (0, 2) is mostly used to monitor damage in steel pipes [7]. Owing to the dispersive propagation and multimodal behavior of L (0, 2), the received signal is usually degraded and noisy, making its implementation and location challenging [9,10]. The fundamental torsional guided wave T (0, 1) can be used to facilitate the time domain separation and reduce dispersion for defect detection because T (0, 1) mode is low attenuation, non-dispersive, and the fastest of torsional waves [11]. It has been able to identify and judge pipeline defects, including circumferential cracks [12,13,14]. Dema [15] quantitatively studied the influence of defects by T (0, 1) mode in the frequency range of 10–300 KHz on pipeline reflection, and made finite element prediction through selected examples. The results show that the reflection coefficient of axisymmetric cracks increases monotonically with depth. Houman [16] proposed a method using the power spectrum difference between torsional waves and flexural waves to detect the torsional waves and determine the defect location. Kim and Park [17,18] used the basic torsional mode to characterize the axial and inclined defects in the pipe and found that at a fixed depth of the defect, the reflection coefficient is a linear function of the ratio of the equivalent circumference range of the defect to the outer circumference of the pipe, and is almost independent of the axial range. They further quantitatively studied the interaction of the T (0, 1) torsional mode with axial and oblique defects in a pipe using a mode decomposition technique to separate the multimodal signals reflected from the defects, and found that the reflection coefficient of an axial crack initially increases with the crack length, but finally reaches an oscillating regime. Gu Tao et al. [19] used torsional guided waves to monitor bent pipes and concluded that the defects on the inner side of the arch back are more difficult to detect than those on the outer side of the arch back. Yeung [20] presented a computationally efficient time-domain spectral finite element method (SFEM) that couples torsional and flexural motions of guided waves to take into account guided wave propagation in pipes. He found that the torsional guided wave propagation, scattering, and mode conversion can be predicted by the SFEM accurately. The application and development of torsional guided waves are later than that of longitudinal guided waves because their excitation methods in structures are more complex than longitudinal guided waves [21].

Circumferential defects cause a series of hidden dangers, such as leakage of transport medium and pipeline failure, which may lead to major disasters [22,23]; therefore, the monitoring of circumferential defects in pipelines is in great demand. Although torsional guided waves have been applied in research on pipeline defects, more qualitative research or axial locating of defects has been undertaken. Very few studies have been conducted on monitoring the circumferential location of defects in pipelines. The reason is that the exciting method of axisymmetric torsional guided waves in the pipeline is mostly using Electromagnetic Acoustic Transducers (EMATs) in which the transducer is an integral circular ring set on the outer of the pipe [21,24], it is difficult to determine the circumferential location and coverage of a defect because the recorded signals do not provide circumferential information [25]. Recently, some scholars have successfully used PZT to excite a fully axisymmetric torsional wave. Li et al. from Peking University successfully excited completely axis symmetrical torsional mode waves in aluminum tubes with 24 face shear d24 PZT elements excited at the frequency of 150 kHz [26,27]. Then, they used T (0, 1) to detect the defect in the pipe with a through-thickness notch and obtain the axial location of the defect by reflected wave [14]. The demand for monitoring the circumferential location of defects is expected to be met by the distributed PZT elements. The authors have conducted a numerical simulation test of applying torsional guided wave T (0, 1) to monitor steel bar damage and concluded that torsional guided wave T (0, 1) is more sensitive to small defects and more accurate in axial positioning of defects than the commonly used longitudinal guided waves. Based on the current research, this paper aims to use the torsional guided wave T (0, 1) mode to simulate the identification of circumferential defects and provides a theoretical basis for practical applications. The principal contribution of this paper is the proposed method in the monitoring of the circumferential location and coverage of a defect in the pipeline using a radar map based on torsional guided waves.

The remainder of this paper is structured as follows. Section 2 describes the basic characteristics of the torsional mode guided wave of T (0, 1). Section 3 presents the numerical simulation of monitoring circumferential defects based on T (0, 1), which demonstrates the effectiveness of the proposed method, while Section 4 offers the concluding remarks.

## 2. Basic Characteristics of Torsional Mode Guided Wave of T (0, 1)

In a cylindrical structure, ultrasonic guided waves propagating along its axis mainly have longitudinal, torsional, and bending modes. By observing the dispersion curve and wave structure diagram of the guided wave, the group velocity and phase velocity of different modes at any frequency and the displacement component of the guided wave mode at a specific frequency can be determined [28]. Therefore, it is found that the torsional guided wave T (0, 1) is superior to defect monitoring.

**Figure 1:**
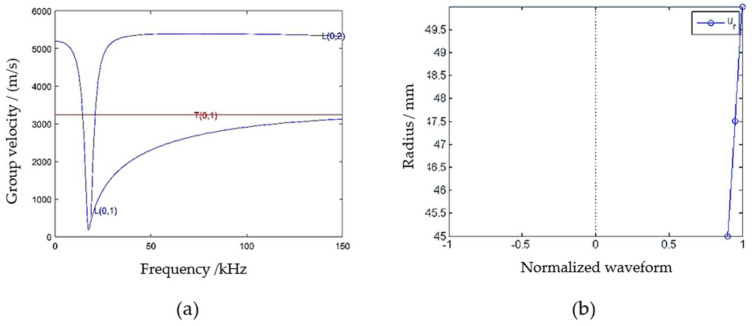
(**a**) Dispersive curves of group velocity in steel pipe with inner diameter of 45 mm and wall thickness of 5 mm; (**b**) structural displacement diagram of torsional guided wave T (0, 1). Note: the dispersive curves and the wave structure diagram of the guided waves are plotted, respectively, by open source program PCDISP and the GUIGUW 2.2 software developed by Professor Alessandro from the University of Bologna in Italy.

Figure 1a shows the dispersive curve of the steel pipe with an inner diameter of 45 mm and a wall thickness of 5 mm, while Figure 1b shows the structural displacement chart of a torsional wave of the rebar at 50 kHz. It can be seen from Figure 1a that the torsional mode of T (0, 1) is non-dispersion; that is, the velocity does not change with the change of frequency. If the torsional mode of T (0, 1) is used as the monitoring technology, the excited guided wave will be single due to the non-dispersive characteristic of T (0, 1), which will obtain a smaller error and greater precision in the position identification of defects than the monitoring method using the mode of L (0, 1). Per Figure 1b, the torsional mode only has circumferential displacement, and the maximum displacement is on the surface, seemingly more sensitive to the surface defect monitoring of the steel pipe than the longitudinal guided wave, such that the defect of pipe can be monitored by placing the probes on the outer surface of the pipe in practical cases. Based on the above characteristics, the damage monitoring of steel pipe using torsional mode will be studied and sensitivity comparison with longitudinal mode wave will be also analyzed in this paper.

## 3. Numerical Simulation of Locating Circumferential Defect in Pipeline Based on T (0, 1)

Because the cross-sectional area of the steel pipe is much larger than that of the steel bar, the locating of the defects on the steel pipe surface is also more complicated than that of the steel bar. In the study of pipeline defects, it is not only necessary to know the axial position of the defect in the pipeline but also to locate its position on the circumference. Relevant studies have shown that the axial position of a pipeline can be determined by the time difference of the guided wave defect echo. This paper focuses on the circumferential location and coverage of defects in the pipeline.

### 3.1. Circumferential Positioning of Defects on the Outer Surface of Pipeline

**Figure 2:**
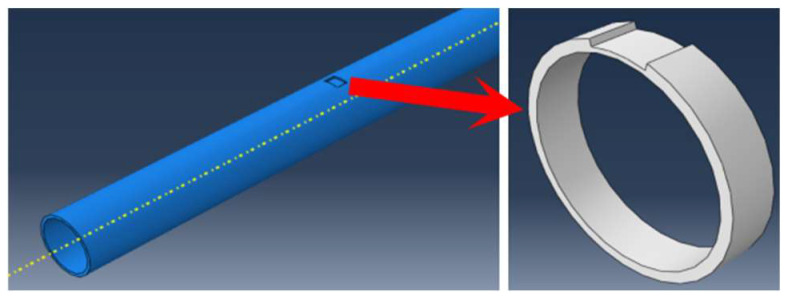
Schematic diagram of pipeline defects.

**Figure 3:**
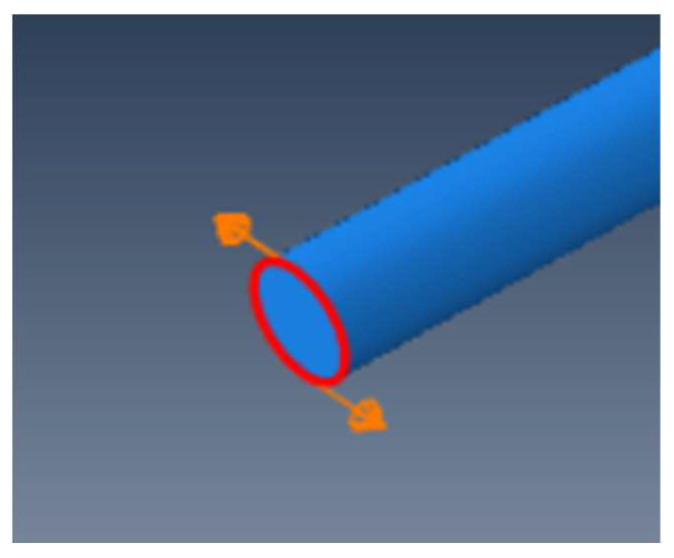
Schematic diagram of guided wave excitation of T (0, 1).

**Figure 4:**
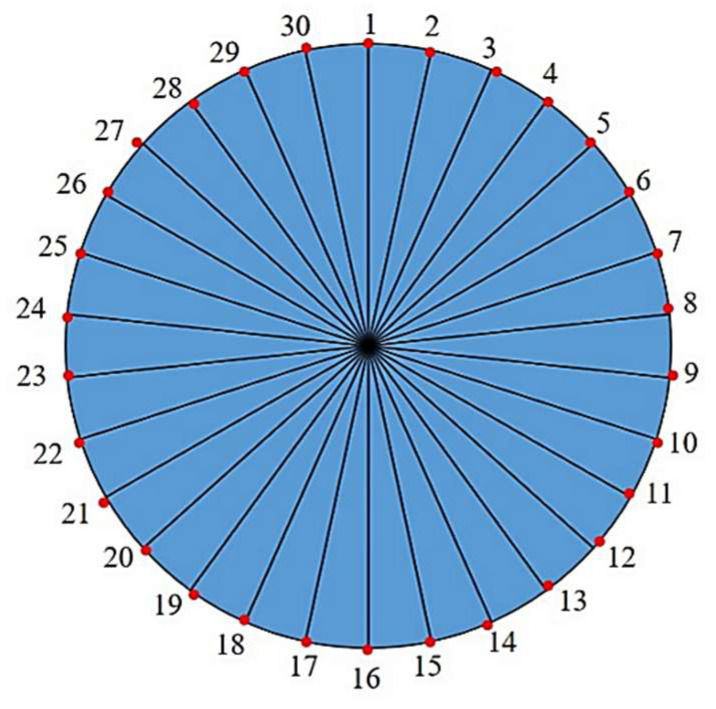
Schematic diagram of pipeline signal extraction.

As shown in the numerical modeling diagram in Figure 2, a 1 m long pipeline model with an inner diameter of 45 mm and an outer diameter of 50 mm is built using ABAQUS. Set the material parameters at elastic modulus E = 208.3 GPa, density ρ = 7700 kg/m^3^, and Poisson’s ratio μ = 0.29, and made the groove defect in the middle of the outer surface of the upper part of the pipeline with the defect depth of 3 mm, length of 2 cm and the width as the arc length corresponding to 30°. Eight node hexahedral elements are selected to divide the steel pipe, and tetrahedral elements are used to refine the defects. The corresponding dispersion curve is shown in the Figure 1a. It can be found from the dispersion curve that the torsional mode T (0, 1) is a non-dispersive mode, which is the mode selected for monitoring. In actual operations, the excitation frequency of torsional mode should be controlled between the cut-off frequencies of L (0, 1) low pass and L (0, 2) high pass. However, only a simple torsional mode is excited in the monitoring without the interference of the longitudinal mode in the numerical simulation. Given the fact that the lower the guided wave excitation frequency, the longer the excitation bandwidth (which is likely to cause the superposition of modes in the monitoring), the windowed five-peak-wave Hanning signal is selected to excite the guided waves with the excitation center frequency of 50 kHz. Set the right side of the pipeline as fixed and the left side as free, and load the instantaneous circumferential displacement at the left end to simulate the excitation of torsional guided wave as shown in Figure 3. The pipeline section is divided into 30 parts, and 30 signal extraction points are set at the left quarter of the pipeline; that is, the included angle between each two points is 12°. The schematic diagram of signal extraction is shown in Figure 4.

**Figure 5:**
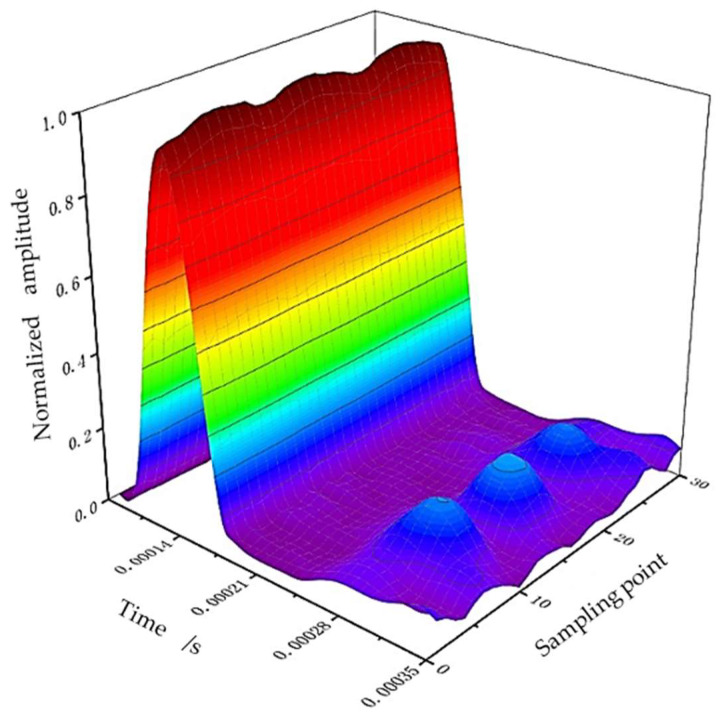
Three-dimensional map of signals extracted at each sampling point.

**Figure 6:**
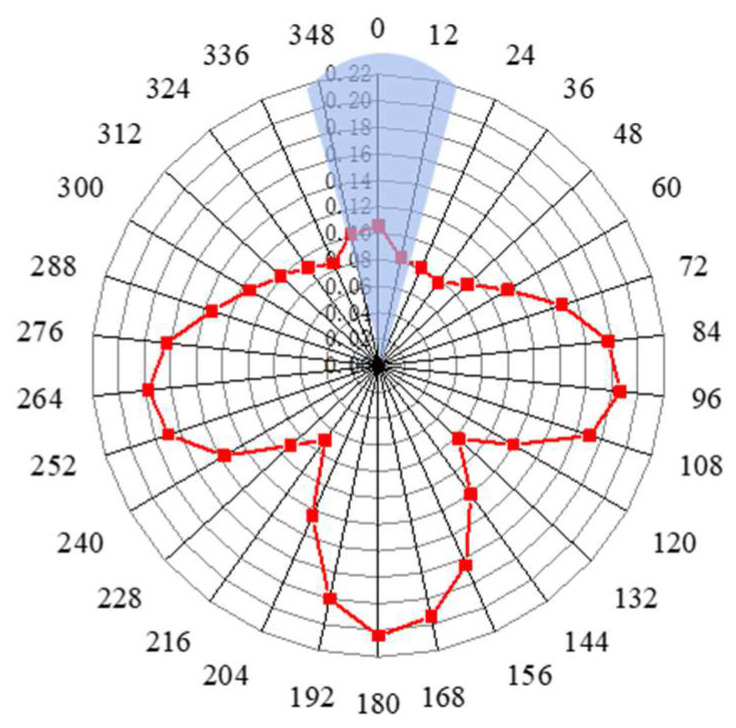
Radar map for circumferential position signals with surface defect depth of 3 mm.

The automatic integration time step size provided by ABAQUS is adopted in the numerical simulation analysis to ensure the convergence of the calculation, and the signals extracted from 30 positions are processed using Hilbert transformation. Hilbert transformation processing can map the signal in the time domain to another time domain by mathematical processing, such that the characteristic information can be highlighted, and the amplitude of the guided wave signal and its corresponding time can be found clearly and intuitively, which is conducive to defect location and damage analysis [29]. Then, a three-dimensional surface graph based on the obtained data after defining the instantaneous envelope at any time has been created, as shown in Figure 5. The peaks of the direct wave and defect echo can be clearly observed in the figure. At the same time, it can be found that the wave packets of defect echo are not simply connected together, but there are roughly three wave packets around these 30 sampling positions. In order to observe the relationship of these echoes more intuitively, the maximum value at the echo position of each defect has been extracted, drawn in the form of a radar diagram, and expressed in the form of a circular section, as shown in Figure 6.

### 3.2. Comparative Analysis of Circumferential Positioning of Defects in Different Parts

**Figure 7:**
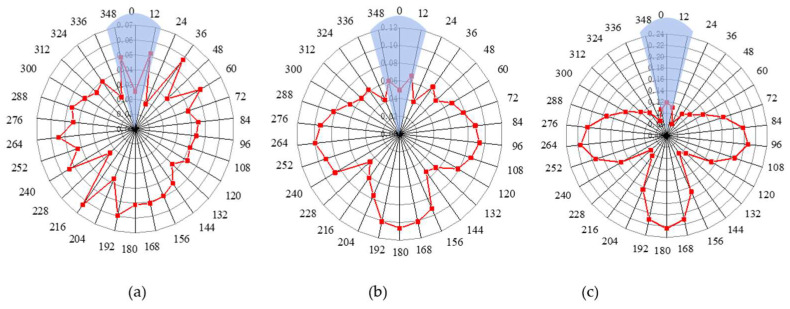
Circumferential positioning diagram of different defect depths on inner wall of pipeline: (**a**) defect depth of 1 mm; (**b**) defect depth of 2 mm; (**c**) defect depth of 4 mm.

A similar pipeline groove defect is set on the inner wall of the pipeline, with the same size as that of the outer wall. The height of the defect starts from 1 mm and increases in 1 mm increments until 4 mm. The echo signal peaks under different defect depth conditions are extracted and drawn using a radar chart. Figure 7 shows the circumferential positioning diagram for different defect depths when the defect is on the inner wall.

It can be seen that when the depth of the defect is only 1 mm, the circumferential position of the defect cannot be accurately located because the radar diagram shows a disordered law. However, when the defect depth gradually deepens, the pattern also becomes regular. The results presented in the radar chart are consistent with that when the defect is on the outer surface; that is, it is a ‘T’ shape on the whole, and the actual position of the defect is directly above the ‘T’ shape, hence the circumferential position of the defect on the inner wall of the pipeline can also be located. And the greater the defect depth, the amplitude of the three ends corresponding to the ‘T’ shape also increases.

**Figure 8:**
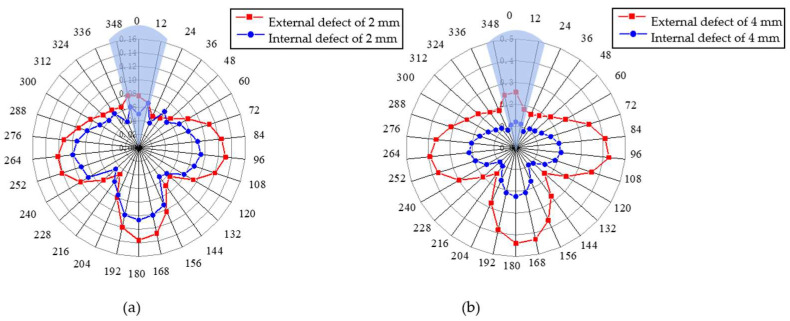
Radar map of circumferential positioning of internal and external defects of pipeline with different defect depths: (**a**) defect depth of 2 mm; (**b**) defect depth of 4 mm.

The defect depth is set as 2 mm and 4 mm, respectively, and the characterization results of the radar diagram are compared when the defect is located on the outer and inner walls of the pipeline, as shown in Figure 8. It can be observed from the figure that under the two defect depth conditions, the radar diagram shows a clear ‘T’ shape and the amplitude of the defect on the outer wall is greater than that on the inner wall of the pipeline. The greater the depth, the greater the amplitude difference between the two conditions. The results are consistent with the waveform structure of the torsional guided wave.

**Figure 9:**
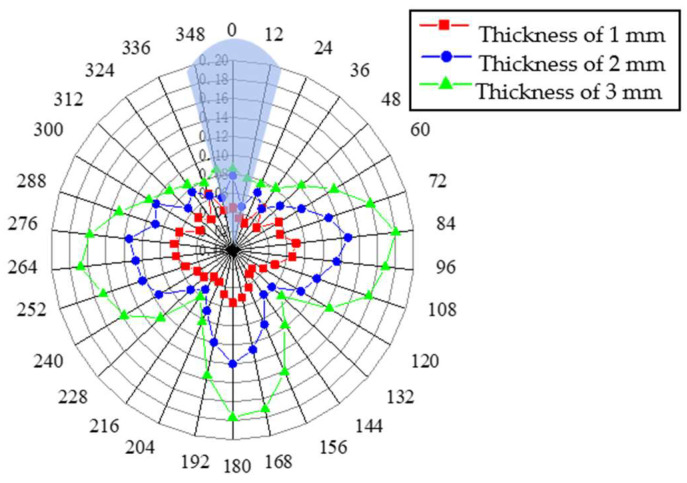
Radar map of circumferential positioning of internal defects in pipe wall thickness.

Figure 9 shows the results of the circumferential positioning radar with different defects located inside the pipe wall. It can be seen that when the defect is located inside the pipe wall, the results also follow the same law as above. Under the thickness of three different defects, the radar diagram always shows a ‘T’ shape, and the amplitude increases with increasing defect thickness.

It can be concluded from the above that for a defect with a defect angle of 30°, regardless of its location in the pipeline, a ‘T’ shape is always shown in the defect circumferential positioning radar diagram by the numerical simulation. When the defect depth is small, such as the defect thickness is only 1 mm, the circumferential position cannot be judged accurately. However, with the increase in defect thickness, the amplitude of the signal increases accordingly, and the ‘T’ shape becomes clear in the monitoring result, and the defect is located directly above the ‘T’ shape.

### 3.3. Comparative Analysis of Circumferential Positioning Results of Defects by Torsional Wave and Longitudinal Wave

The pipeline model in the above section is established using ABAQUS 2020 software with the defect angle set at 30° and the defects located on the inner and outer walls of the pipeline, respectively. The numerical simulation of pipeline monitoring is performed using the excitation mode of torsional guided wave T (0, 1) and longitudinal wave L (0, 2), and the circumferential positioning results of defects under two different monitoring modes are compared.

**Figure 10:**
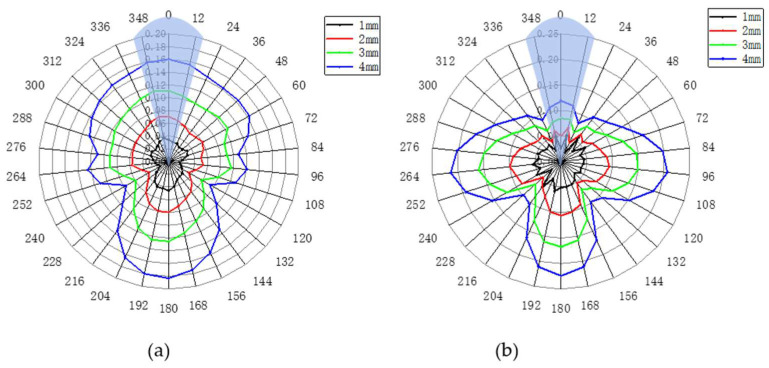
Radar map of circumferential location of defects in two monitoring methods under the condition of internal pipe defects: (**a**) longitudinal wave L (0, 2) monitoring method; (**b**) torsional wave T (0, 1) monitoring method.

Figure 10 shows the radar diagram of defect circumferential positioning using the two monitoring methods when the defect is in the pipe wall. The blue shadow represents the actual location of the defect. It can be seen that when the longitudinal wave L (0, 2) method is used, the final radar diagram cannot be represented by a clear image and the actual position of the defect cannot be effectively located. In contrast, when the torsional wave method is used with an increase in defect depth, the ‘T’ shape of the final radar chart becomes clearer and more obvious, and the circumferential position of the defect can be well judged. Meanwhile, it can be seen from the comparison of the echo amplitude that the maximum reflectivity of the torsional wave exceeds 40%, while that of the longitudinal wave does not exceed 25%, which also shows that the torsional wave can better locate the circumferential position of defects.

It can be seen from the defect circumferential positioning radar chart in Figure 9 that the monitoring method using torsional guided wave T (0, 1) can better locate the circumferential position of the defect than the longitudinal wave L (0, 2) method, regardless of the result graph presented by the circumferential positioning radar chart or the echo amplitude of the defect, showing the superiority of torsional guided wave T (0, 1) in defect monitoring.

### 3.4. Determination of the Circumferential Coverage of a Defect in the Pipeline

**Figure 11:**
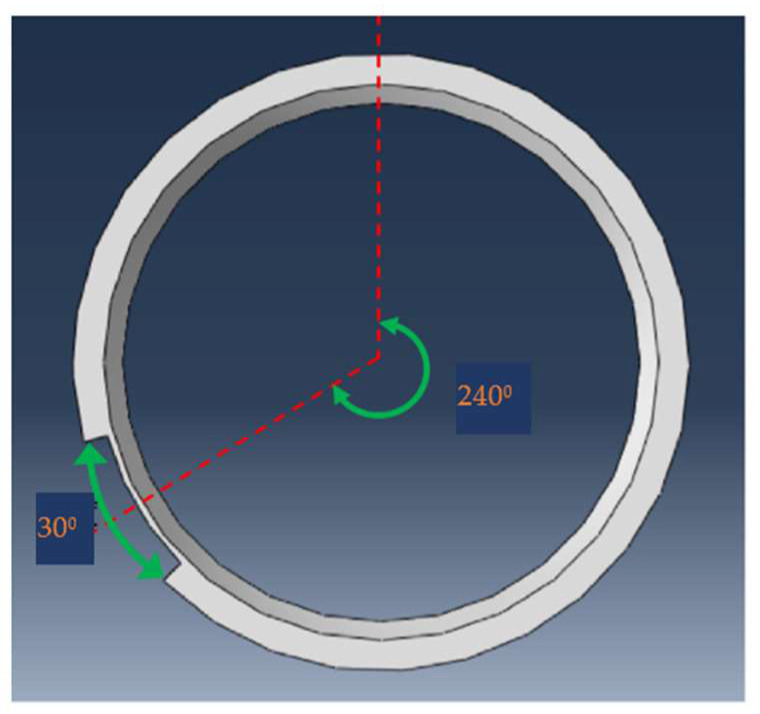
Schematic diagram of pipeline defect angle.

A pipeline model diagram with surface groove defects is established to study the influence of different defect angles on the monitoring. A schematic diagram of the pipeline defects is shown in Figure 11, with a pipe wall thickness of 5 mm, defect depth of 4 mm, and center angle of the defect of 240° (obtained by rotating clockwise with the 12 o’clock direction of the circumference as the starting point). The defect angle is centered at 240° and expands to both sides. It starts at 30° and increases by 30° until the maximum defect angle is 180°.

The signal value is similarly extracted from the left quarter of the pipeline. Fifteen signal extraction points are set on one circle of the circumference and the received signals are transformed using Hilbert transformation. Extreme values of the defect echo are extracted and drawn on a radar map.

**Figure 12:**
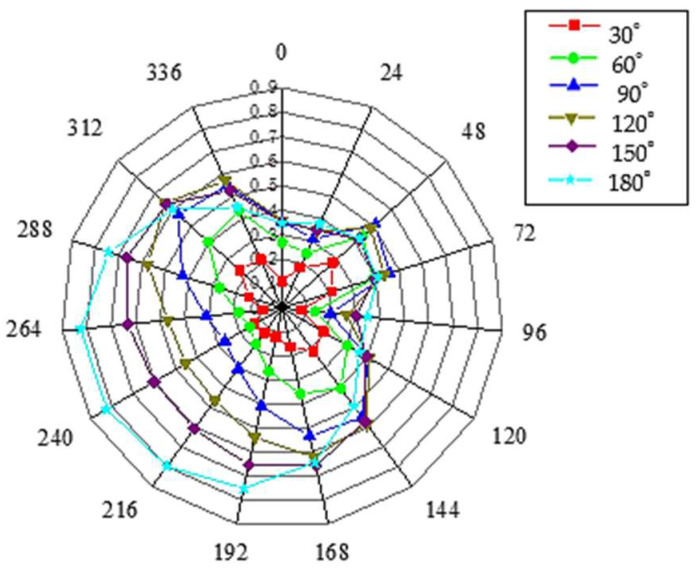
Circumferential positioning radar map with different defect angles.

A circumferential positioning radar diagram for different defect angles is shown in Figure 12. It can be seen that when the defect angle is below 90°, the graph result shows a ‘T’ shape, with 240° of the defect center as the symmetry axis, and the defect position is above the ‘T’ shape, which is consistent with the previous conclusion. When the defect angle is greater than 90°, the amplitude of the defect echo at the defect position begins to increase gradually, and the top of the ‘T’ shape begins to bulge, while echo amplitude at the non-defect position does not change significantly with the increase in defect angles.

**Figure 13:**
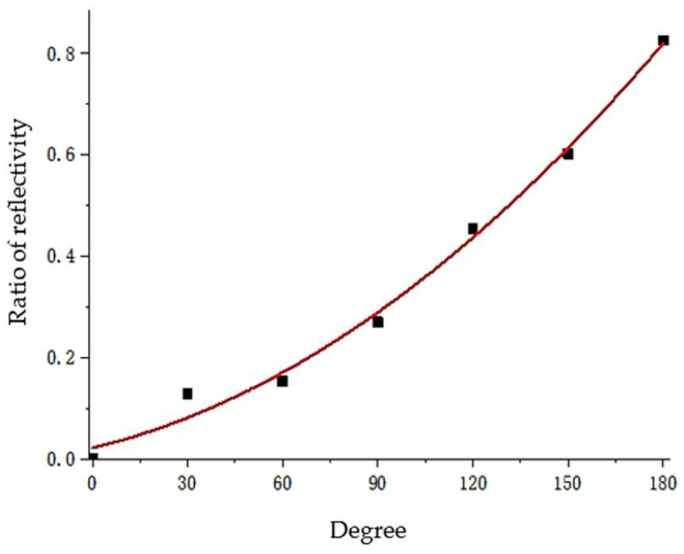
Preliminary linear fitting image of pipeline defect angle judgment.

By studying the changing trend of the result diagram, the signal amplitude corresponding to the defect center (240°) is selected for linear fitting to determine the arbitrary defect angle value of the groove on the pipeline surface. A preliminary linear-fitting image is shown in Figure 13. It can be seen that when the defect angle is less than 60°, there is a certain error in the fitted curve, an error that easily occurs during the extraction process when the amplitude of the extracted signal echo is also small with a small defect. Given that the defect angle cannot be well characterized by only two data points, it is decided to add fitting data points in the range of 0 to 60°. For the pipeline defect within the range of 60° defect angle, six groups of simulation experiments were set up in the increment of 10° defect angle to extract the echo amplitude at the defect position again and perform linear fitting to further optimize the fitting formula. The final results are as follows:

(1) $$\left\{ \begin{array}{l} y=1.6667\times 10^{-6}x^{3}-1.4792\times 10^{-4}x^{2}+0.00803x-0.00223(0\leq x<60)\\ y=1.66209\times 10^{-5}x^{2}+0.00151x+0.02156(60\leq x<180) \end{array}\right.$$

where *y* is the defect echo amplitude and *x* is the defect angle value.

To verify the obtained formula, five groups of working conditions with defect angles of 24°, 48°, 56°, 100°, and 137° were set, respectively, and the defect echo amplitude *y* at the defect obtained from the numerical simulation was substituted into the formula to obtain the defect angle value. The results are shown in Table 1.

It can be seen from the verification results that the linear fitting formula has a very high accuracy in judging defects with larger angles. When the defects are 100° and 137°, the judgment error is no more than 1%, while when the defect angle is less than 60°, the judgment error of the optimized linear fitting formula on the defect angle is no more than 4%. It is proved that the echo amplitude at the defect position of the pipeline can be extracted for linear fitting, which can be used to quantitatively judge the circumferential coverage of a defect on the outer surface of the pipeline.

## 4. Conclusions

In this paper, a pipeline is selected as the research object for the numerical simulation of damage monitoring. The response of torsional waves to defects in pipelines at various locations, depths, and angles is examined, and the monitoring outcomes are compared with those of longitudinal waves. The results lead to the following conclusions.

(1) To determine the circumferential positioning of the pipeline, 30 signal extraction points are established on the circumferential surface. The extracted defect echo amplitude is then projected onto a radar map. The findings demonstrate that for a defect with an angle of 30°, whether it is located inside the pipe wall, outside the pipeline, or inside the pipe wall, the circumferential positioning radar map of the defect is always accompanied by a ‘T’ shape, and the defect is located directly above the ‘T’ shape, which can be used to locate and analyze the circumferential defect of the pipeline, whether it is located inside or outside the pipeline or inside the pipe wall.

(2) To determine the circumferential position of defects, the torsional and longitudinal waves are compared and examined. Regardless of the circumferential positioning radar map result graphics or the echo amplitude of defects, the results demonstrate that the torsional guided wave monitoring method can better locate the circumferential position of defects than the longitudinal wave method. The benefits of torsional guided waves in defect monitoring are clearly demonstrated.

(3) Extracting the echo amplitude at the defect point of the pipeline for linear fitting can be utilized to quantitatively assess the circumferential coverage of a defect on the outer surface of the pipeline. The highest allowed judgment error is less than 4%, and the verification is highly accurate.

Moreover, this study was performed as the first step. With the T (0, 1) being successfully excited by PZT by some researchers recently, the lab experiment and in-field application for the coated pipelines will be carried out in the following step to achieve a comprehensive understanding of damage monitoring.

## Figures and Tables

**Table 1:** Angle verification of arbitrary defects.

Actual Defect Angle	Numerical Simulation Reflectance	Corresponding Defect Angle	Error/%
24	0.1191	23.24	3.17
48	0.1683	46.25	3.65
56	0.1854	54.60	2.5
100	0.3398	101.00	0.99
137	0.5309	136.66	0.29

## Data Availability

Not applicable.
